# Treating to target in psoriatic arthritis: assessing real-world outcomes and optimising therapeutic strategy for adults with psoriatic arthritis—study protocol for the MONITOR-PsA study, a trials within cohorts study design

**DOI:** 10.1186/s13063-021-05142-7

**Published:** 2021-03-04

**Authors:** Ines Rombach, William Tillett, Deepak Jadon, Laura Tucker, Marion Watson, Anne Francis, Yvonne Sinomati, Lucy Eldridge, Melina Dritsaki, Susan J. Dutton, Hussein Al-Mossawi, Nicola Gullick, Ben Thompson, Laura C. Coates

**Affiliations:** 1 Oxford Clinical Trials Research Unit, Nuffield Department of Orthopaedics, Rheumatology and Musculoskeletal Sciences, University of Oxford, Oxford, UK; 2 Royal National Hospital for Rheumatic Diseases, Bath, UK; 3 Department of Pharmacy and Pharmacology, University of Bath, Bath, UK; 4 Department of Medicine, University of Cambridge, Cambridge, UK; 5 Nuffield Department of Orthopaedics, Rheumatology and Musculoskeletal Sciences, University of Oxford, Oxford, UK; 6 Rheumatology Department, University Hospitals Coventry and Warwickshire NHS Trust, Coventry, UK; 7 Department of Rheumatology, Musculoskeletal Unit, Newcastle-upon-Tyne Hospitals NHS Trust, Newcastle upon Tyne, UK

**Keywords:** Psoriatic arthritis, Treat-to-target, TWiCs, Trials within cohorts, Cohort multiple RCT or cmRCT

## Abstract

**Background:**

The Tight Control of psoriatic arthritis (TICOPA) trial confirmed improved clinical outcomes with a treat to target (T2T) strategy in psoriatic arthritis (PsA). This consisted of 4-weekly review and escalation of ‘step up’ therapy (single disease modifying therapy (DMARD), combination DMARDs and then biologics) based on remission criteria. Based on this, a T2T approach is supported by European PsA treatment recommendations. However, it is not commonly implemented in routine care primarily due to feasibility and cost concerns. In the TICOPA trial, the same treatment regime was used for all participants regardless of their disease profile. Despite the recognition of PsA as a highly heterogeneous condition, no studies have tailored which drugs are used depending on disease severity. The cohort will establish real world outcomes for the T2T approach in PsA and also form the basis of a trials within cohorts (TWiCs) design to test alternative therapeutic approaches within embedded clinical trials providing an evidence base for treatment strategy in PsA.

**Methods:**

The Multicentre Observational Initiative in Treat to target Outcomes in Psoriatic Arthritis (MONITOR-PsA) cohort will apply a T2T approach within routine care. It will recruit newly diagnosed adult patients with PsA starting systemic therapies. The cohort is observational allowing routine therapeutic care within NHS clinics but a T2T approach will be supported when monitoring treatment within the cohort. Eligible participants will be adults (≥18 years) with active PsA with ≥ 1 tender or swollen joints or enthesis who have not previously had treatment with DMARDs for articular disease.

**Discussion:**

This study is the first TWiC designed to support a fully powered randomised drug trial. The results from the observational cohort will be compared with those observed in the TICOPA trial investigating the clinical effectiveness and health care costs of the pragmatic T2T approach. Nested trials will provide definitive RCT evidence establishing the optimal management of PsA within the T2T approach. The TWiCs design allows robust generalizability to routine healthcare, avoids disappointment bias, aids recruitment and in future will allow assessment of longer-term outcomes.

**Trial registration:**

ClinicalTrials.gov NCT03531073. Retrospectively registered on 21 May 2018.

## Administrative information

Note: the numbers in curly brackets in this protocol refer to SPIRIT checklist item numbers. The order of the items has been modified to group similar items (see http://www.equator-network.org/reporting-guidelines/spirit-2013-statement-defining-standard-protocol-items-for-clinical-trials/).

**Table:** 

Title {1}	A multicentre observational psoriatic arthritis cohort study addressing real-life outcomes of a treat to target approach in routine clinical practice.
Trial registration {2a and 2b}.	NCT03531073 https://clinicaltrials.gov/ct2/show/NCT03531073 retrospectively registered 21 May 2018.
Protocol version {3}	V6.0 28Jul2020
Funding {4}	Funded by National Institute for Health Research CS-2016-16-016
Author details {5a}	Ines Rombach, Oxford Clinical Trials Research Unit, Nuffield Department of Orthopaedics, Rheumatology and Musculoskeletal Sciences, University of Oxford, UK William Tillett, Royal National Hospital for Rheumatic Diseases, Bath, UK, Department of Department of Pharmacy and Pharmacology, University of Bath, UK Deepak Jadon, Department of Medicine, University of Cambridge, Cambridge, UK Laura Tucker, Nuffield Department of Orthopaedics, Rheumatology and Musculoskeletal Sciences, University of Oxford, UK Marion Watson, Nuffield Department of Orthopaedics, Rheumatology and Musculoskeletal Sciences, University of Oxford, UK Anne Francis, Nuffield Department of Orthopaedics, Rheumatology and Musculoskeletal Sciences, University of Oxford, UK Yvonne Sinomati, Nuffield Department of Orthopaedics, Rheumatology and Musculoskeletal Sciences, University of Oxford, UK Lucy Eldridge, Oxford Clinical Trials Research Unit, Nuffield Department of Orthopaedics, Rheumatology and Musculoskeletal Sciences, University of Oxford, UK. Melina Dritsaki, Nuffield Department of Orthopaedics, Rheumatology and Musculoskeletal Sciences, University of Oxford, UK. Susan J Dutton, Oxford Clinical Trial Research Unit, Nuffield Department of Orthopaedics, Rheumatology and Musculoskeletal Sciences, University of Oxford, UK. Hussein Al-Mossawi, Nuffield Department of Orthopaedics, Rheumatology and Musculoskeletal Sciences, University of Oxford, UK. Nicola Gullick, Rheumatology Department, University Hospitals Coventry and Warwickshire NHS Trust, Coventry, United Kingdom. Ben Thompson, Department of Rheumatology, Musculoskeletal Unit, Newcastle-upon-Tyne Hospitals NHS Trust, UK Laura Coates, Nuffield Department of Orthopaedics, Rheumatology and Musculoskeletal Sciences, University of Oxford and Oxford Biomedical Research Centre, Oxford University Hospitals Trust, Oxford, UK.
Name and contact information for the trial sponsor {5b}	University of Oxford Clinical Trials and Research Governance, Boundary Brook House, Churchill Drive, Headington, OXON, OX3 7LQ Email: ctrg@admin.ox.ac.uk
Role of sponsor {5c}	The study sponsor provided minor input into study design and oversees data collection, management and analysis of study data. The funder has no role in study design but will review any publications prior to publication.

## Introduction

### Background and rationale {6a}

Psoriatic arthritis (PsA) is an inflammatory arthritis estimated to occur in 15% of people with psoriasis [1] affecting around 150,000 people in the UK [2]. Two thirds of people with PsA suffer progressive joint damage with associated disability [3, 4]. People with PsA have similar functional and quality of life impairment to rheumatoid arthritis (RA) [5].

The TICOPA trial was the first randomised controlled trial to demonstrate improved clinical and patient-reported outcomes with a ‘treat to target’ approach in PsA and consisted of 4-weekly reviews and escalation of treatment aiming for the minimal disease activity (MDA) criteria [6]. This led to the 2015 European League Against Rheumatism (EULAR) Treatment recommendations for PsA incorporating as its first recommendation that ‘treatment should be aimed at reaching the target of remission or, alternatively, minimal/low disease activity, by regular monitoring and appropriate adjustment of therapy’ [7]. However, despite the evidence [6] and the EULAR recommendations supporting ‘treat to target’ in PsA [7], it has not been widely implemented [8]. In clinical practice, the 4-weekly review used in the TICOPA trial is not affordable or feasible and is too intensive for both patients starting new therapies or those stable in MDA. We therefore aim to establish a pragmatic feasible ‘treat to target’ approach in a real-life clinic population which can provide similar clinical and health-related quality of life outcomes to those seen in TICOPA at a lower cost. Dissemination of these results will aid translation of ‘treat to target’ into clinical practice.

Within a treat to target (T2T) approach, there is little evidence base to support the choice and sequencing of disease-modifying anti-rheumatic drugs (DMARDs), either conventional (csDMARD) or biologic (bDMARD). Despite the recognition that PsA is a heterogeneous disease, most physicians apply the same ‘step up’ therapy to all patients using single csDMARDs, then combinations of these csDMARDs and then bDMARDs as patients fail to respond to the previous treatment step [7, 9]. For this reason, the tight control algorithm in the TICOPA trial used this approach: first methotrexate, then combination csDMARDs and then potentially escalating to bDMARDs. For patients with mild disease, this may lead to overtreatment and unnecessary side effects as they may not require regular csDMARDs [10]. Although in the early Spondyloarthritis (SpA) study from Leeds, only 4 of 59 patients had PsA, a previous study in undifferentiated peripheral SpA found that 55% of patients did not require csDMARDs and could be managed with only intra-articular steroid injections and analgesia [10].

At the other end of the spectrum, patients with severe disease may benefit from more aggressive early intervention [11]. Both the Group for Research and Assessment of Psoriasis and Psoriatic Arthritis (GRAPPA) and EULAR International treatment recommendations utilise a ‘step up’ approach to treatment [7, 9] but suggest more intensive therapy for those with poor prognostic factors based on expert opinion. Applying initial intensive therapy has shown improved outcomes in other inflammatory arthritides such as RA [12] but has never been tried in PsA. Combinations of DMARDs have shown some superiority over single therapies in PsA [13] but the data are limited. Early use of tumour necrosis factor (TNF) inhibitors has also been shown to be superior to methotrexate for patients with more severe disease [11, 14] but is more costly. If an early course of TNF inhibitors can rapidly suppress inflammation allowing treatment to be withdrawn and response maintained on methotrexate, this may be a cost-effective model for early use.

Given this unmet need to address treatment strategy in PsA within the T2T approach, the cohort has been designed to support future pragmatic treatment studies.

### Objectives {7}

The objective of the Multicentre Observational Initiative in Treat to target Outcomes in Psoriatic Arthritis (MONITOR-PsA) cohort is to assess clinical and patient-reported outcomes, as well as costs associated with a pragmatic routine implementation of a T2T approach, and compare those to the outcomes of the TICOPA trial.

The MONITOR-PsA cohort also aims to facilitate recruitment to a number of embedded trials (trials within cohorts (TWiCs) design) to test alternative therapeutic approaches in embedded clinical trials to provide an evidence base for treatment strategy in PsA.

### Trial design {8}

The MONITOR-PsA cohort establishes an inception cohort of PsA patients receiving a T2T treatment approach within routine care. Given that this programme aims to address outcomes with a pragmatic ‘T2T’ approach in a real-life cohort and compare other therapeutic interventions, the cohort utilises a Trials Within Cohorts (TWiCs) design [15]. This design is particularly suited to open label trials with ‘treatment as usual’ as the comparator. It is ideal for chronic conditions, situations where multiple trials may be performed and where expensive desirable treatments are being trialled [15]. This method recruits a central cohort having ‘treatment as usual’ with regular observations and then adds pragmatic trials of alternative therapies where a random group of eligible patients are selected. This allows robust generalizability from studies to routine health care, avoids attrition and disappointment bias from controls in open label studies as patients only receive information relevant to their care, aids recruitment to trials, allows routine collection of long-term outcomes and increases efficiency with multiple trials within one cohort [15].

To date, one small feasibility study has used a TWiCs design within a drug trial [16], but no large powered drug trials have used this design. Initially, there were two interventional studies planned within the MONITOR-PsA cohort but the hope is that this cohort will support multiple effectiveness trials comparing different approaches with the standard ‘step-up’ care model that is most commonly used in routine practice in PsA. These can offer alternative initial therapy at the time of diagnosis or can be integrated at a defined time point in the treatment pathway, for example, choosing a first biologic DMARD or failing a first biologic DMARD. All of these studies would offer interventional arms which will then be compared to the standard step up approach used in the cohort. Patient research partners have been involved in the study concept and design from the beginning of the process. They supported the idea of utilising this new efficient study design and have had significant input into the outcome measures collected within the MONITOR-PsA cohort. Patient research partners continue to advise on the management of the cohort and the design of embedded trials.

## Methods: participants, interventions and outcomes

### Study setting {9}

The MONITOR-PsA cohort is observational with implementation of a feasible treat to target approach, currently running within the rheumatology departments of four secondary care hospitals in the UK.

### Eligibility criteria {10}

Eligible participants will be as follows: willing and able to give informed consent for the study; male or female; 18 years or over; have a clinical diagnosis of PsA confirmed by the ClAsSification of Psoratic ARthritis (CASPAR) criteria [17]; active PsA with ≥ 1 tender or swollen joints or enthesis; not previously had treatment with DMARDs for articular disease; and, in the investigators’ opinion, is able and willing to comply with all study requirements. All potentially eligible participants will be approached for participation in the study by their treating rheumatology team.

Female participants of child bearing potential and male participants whose partner is of child bearing potential must be willing to ensure that they or their partner use effective contraception during potentially teratogenic DMARD treatment and for 3 months thereafter (or 2 years if received leflunomide unless treated with washout therapy) as in standard practice. Women who are pregnant, nursing or planning pregnancy during the following 12 months will only receive DMARDs that are appropriate according to British Society of Rheumatology (BSR) recommendations.

Participants may not enter the study if any of the following apply: Current or previous treatment of arthritis with synthetic DMARDs (including methotrexate, leflunomide or sulfasalazine) or biologic DMARDs (including TNF, IL12/23 or IL17 inhibitor therapies) or targeted synthetic DMARDs (PDE4 of JAK inhibitor therapies); or use of investigational therapies within 1 month or 5 biological half-lives of the baseline study visit (whichever is longer).

### Who will take informed consent? {26a}

Potential participants will be identified from new referrals to the rheumatology service, particularly those referred to the early arthritis clinic or dedicated PsA clinic. Patients will be identified and approached initially by their clinical care team and will be given brief information about the study. If interested, a full patient information sheet (PIS) and informed consent form (ICF) will be given to them and explained. If they are willing to participate in the observational study, consent can be obtained on the same day. However, they will be allowed as much time as they wish to consider their participation. Written informed consent will then be obtained by means of participant dated signature and dated signature of the person who presented and obtained the Informed Consent. The person who obtained the consent must be suitably qualified and experienced and have been authorised to do so by the chief/principal investigator (CI/PI).

Given the TWiCs design which this cohort underpins, the consent for the cohort includes an optional consent to use participants’ personal details to contact them about future research and explains that a random selection of those eligible for an intervention arm of an embedded treatment study (including clinical trials of medicines) may be invited to participate in these additional embedded studies. Participants are randomised to the offer of an intervention but obviously do not have to consent to participate in these. If participants consent to being approached about further research, they are made aware that their anonymised data may be used as a ‘control arm’ to compare with additional interventional studies.

### Additional consent provisions for collection and use of participant data and biological specimens {26b}

Additional, optional consent is sought from participants for the collection of biological specimens including blood, urine and stool samples. In addition, the consent form asks for a participant’s consent to share their anonymized data with other research groups.

## Interventions

### Explanation for the choice of comparators {6b}

The MONITOR-PsA cohort will receive ‘treatment as usual’ with regular observations and then additional pragmatic trials of alternative therapies can be implemented where a random group of eligible patients are selected.

### Intervention description {11a}

Within the cohort, patients will be assessed every 12 weeks during the first year of therapy and will be treated in line with current standard step-up care. This will consist of initial therapy with methotrexate alone, then alternative DMARDs for non-response or intolerance, then potentially biologic therapy following NICE recommendations [2].

If an assessment is performed after 12 weeks on a new therapy and participants have reached the target of treatment, then this should be continued. If they have not achieved minimal disease activity criteria (MDA) but have shown significant improvement since starting the new therapy (a reduction in tender and swollen joint counts of at least 20%) then treatment should be continued for a further 12 weeks before a repeat assessment. If they have not responded significantly, then the next step in the treatment protocol should be offered. For biological and targeted synthetic DMARDs, response must be recorded using the PsA Response Criteria (PsARC) to fulfil NICE guidelines on the prescriptions of these therapies. The timing of this assessment varies by drug (TNF inhibitors 12 weeks, apremilast and secukinumab 16 weeks, ustekinumab 24 weeks). If patients do not achieve a PsARC response, then their treatment should not be continued. Providing that new therapies can be tolerated by the patient and do not cause significant toxicity, such that patients are unwilling to continue with treatment or that treatment must be stopped due to safety concerns, a trial of a minimum of 12 weeks therapy should be given before changing or adding an alternative DMARD.

### Criteria for discontinuing or modifying allocated interventions {11b}

The step-up treatment within the cohort is based around assessment of the MDA criteria as these are a validated and recommended target of treatment in PsA. If patients do not achieve MDA by 24 weeks after initiating therapy (or have not had an improvement of at least 20% in active joint counts after 12 weeks on therapy), then treatment should be escalated to the next step.

### Strategies to improve adherence to interventions {11c}

This is an observational study embedded within usual clinical practice. Hospital sites will employ their usual strategies to improve adherence to treatment within the cohort. These include counselling on disease-modifying anti-rheumatic drugs by physicians, specialist nurses and pharmacists, follow-up visits for counselling, adherence and monitoring and written support materials given to patients.

### Relevant concomitant care permitted or prohibited during the trial {11d}

As this is an observational cohort supporting pragmatic trials, there are no prohibited concomitant care or interventions within this study.

### Provisions for post-trial care {30}

Within the MONITOR-PsA cohort, all medications will be prescribed via usual care pathways and patients will be able to continue on their medications at the end of the study as long as they are responding well to treatment. For embedded trials, some medications may not be available beyond the end of the trial period but that will be made clear to participants at the time of consent to the embedded study and alternative therapies will be offered as appropriate in standard rheumatology practice. At the end of any embedded study period, participants return to usual cohort follow-up allowing long-term assessment of the impact of any tested interventions. Insurance is in place via the sponsor and participating NHS sites for any harm resulting from trial participation.

### Outcomes {12}

The primary endpoint is the proportion of patients achieving the PsA Disease Activity Score (PASDAS) ‘good’ response [18] at 48 weeks. The PASDAS is a composite score including both clinical assessment and patient-reported outcomes [19].

PASDAS responses are calculated as follows:

**Table:** 

PASDAS calculation	(((0.18 √ physician global VAS) + (0.159 √ patient global VAS) – (0.253 x √ SF36 − physical component summary score (PCS)) + (0.101 x LN (swollen joint count + 1)) + (0.048 x LN (tender joint count + 1)) + (0.23 x LN (Leeds enthesitis index + 1)) + (0.37 LN (tender dactylitis count + 1)) + (0.102 x LN (C − reactive protein (CRP) + 1)) + 2) x 1.5.		
	Improvement		
Final PASDAS score	> 1.6	≤ 1.6 but > 0.8	≤ 0.8
≤ 3.2	Good	Moderate	Poor
> 3.2 but < 5.4	Moderate	Moderate	Poor
≥ 5.4	Moderate	Poor	Poor

The results will be discussed in the context of the TICOPA trial [6] (*n* = 206) to see if they are consistent. The patients recruited early in the course of the study who therefore have longer-term follow-up data will enable us to explore durability of the response.

Key secondary endpoints include:

- Time from baseline to first achieving MDA [20] and PASDAS good response [18]
- Proportion achieving PASDAS good response [18] at 24 weeks
- Proportion achieving PASDAS moderate response [18] at 24/48 weeks
- Healthcare costs and quality adjusted life years (QALYs) (see below)

Exploratory outcomes (not available in TICOPA dataset) will include:

- Change in PsA Impact of Disease (PsAID) score [21] from baseline to 48 weeks
- Proportion achieving PsAID participant acceptable symptom state (≤ 4) [21] at 48 weeks
- Change in work productivity (WPAI-SHP) [22] (absenteeism, presenteeism and productivity loss) at 24 and 48 weeks
- Side effects reported at each time point, combined with the Treatment Satisfaction Questionnaire for Medication (TSQM)
- Outcomes at time points later than 48 weeks during the longer-term follow-up of the cohort.
- Explore immune response in patients with PsA and how this relates to clinical effectiveness with standard therapies

All outcomes are collected via electronic case report forms (eCRFs). Patient-reported outcomes are inputted directly by the participants using a tablet computer, with a paper version available in case completion on the tablet computer is not possible. A full list of outcome measures and their timings are provided in Table 1.

**Table 1:** Outcome measures in the MONITOR-PsA cohort study

Procedures	Visits							
	Baseline cohort							
	Week 0	Week 12	Week 24	Week 36	Week 48	Week 72	Week 96	Annually (after 96 weeks)
Informed consent	X							
Demographics	X							
Medical history	X	X	X	X	X	X	X	X
Medications	X	X	X	X	X	X	X	X
Physical examination	X	X	X	X	X	X	X	X
Routine blood tests (FBC, U&Es, LFTs, CRP and eGFR)	X	X	X	X	X	X	X	X
Immunology (RF, ACPA, ANA)	X							
Radiographs of hands/feet/spine	X				X		X	X
Eligibility assessment	X							
Adherence		X	X	X	X	X	X	X
68/66 Joint count	X	X	X	X	X	X	X	X
Leeds and SPARCC enthesitis index	X	X	X	X	X	X	X	X
Dactylitis count	X	X	X	X	X	X	X	X
Psoriasis assessment (PASI and BSA)	X	X	X	X	X	X	X	X
Physician VAS	X	X	X	X	X	X	X	X
Metrology with BASMI (if axial involvement)	X				X		X	X
Patient questionnaires (VAS, HAQ, SF36, PsAID, WPAI, BASDAI, BASFI)	X	X	X	X	X	X	X	X
TSQM questionnaire		X	X	X	X	X	X	X
EQ-5D-5L questionnaire	X		X		X	X	X	X
Healthcare utilisation data			X		X	X	X	X
Adverse event assessments	X	X	X	X	X	X	X	X

All adverse events will not be collected in this study as all the medications used are currently used in PsA and have well documented safety profiles. Only adverse events of special interest (either extra-articular manifestations of the disease or those likely to be related to the therapy used for PsA) occurring during the study will be collected at each study visit by patient questionnaire and physician report as below:

- Patient reported: nausea/vomiting; heartburn/dyspepsia; diarrhoea; fatigue; hair loss; and injection site reactions
- Physician reported: infections; liver function test abnormalities; neutropenia/leucopenia; and uveitis.

Serious adverse events that, in the opinion of the CI, were ‘related’ (resulted from administration of any of the research procedures) and ‘unexpected’ in relation to those procedures will be reported.

### Participant timeline {13}

Participants are recruited at or after the time of diagnosis before they start on disease-modifying therapy. Study visits in the cohort will be at 0, 12, 24, 36 and 48 weeks, every 6 months in year 2 and yearly thereafter in line with routine practice to minimise burden and support retention of participants. In case of treatment escalation at a later time point, visits will be repeated at 12, 24 and 48 weeks.

Different embedded trials can be included within this approach either investigating initial treatment (with participants randomised at the MONITOR-PsA baseline visit) or at another specific treatment point in the follow-up period, for example failure of a particular therapy when subsequent treatment is investigated. Study visits for embedded trials will be aligned with those for the cohort wherever possible.

### Sample size {14}

We aim to recruit a minimum of 500 participants to the cohort, which may be extended if additional trials are funded. No formal sample size calculations were performed for the cohort. We estimate that around 200 participants will receive the standard treatment within the cohort, which will allow for robust estimation of the primary outcome, i.e. robust confidence intervals around the proportion of participants receiving a PASDAS good response at 48 weeks, to be compared to the outcomes observed in the TICOPA trial [6].

### Recruitment {15}

All patients newly diagnosed with psoriatic arthritis are invited to join the cohort. The PIs at each site lead on either psoriatic arthritis or early inflammatory arthritis clinics allowing easy identification of the majority of newly diagnosed patients. Initially, the study was established with three sites, but this has been expanded to improve recruitment rates with the addition of further UK sites. To date, the majority of patients have consented to join the study when invited with only a small number of people declining most commonly because of concerns about the duration of appointments with additional questionnaires.

## Assignment of interventions: allocation

### Sequence generation {16a}

All patients entering into the MONITOR-PsA cohort are registered using a centralised service run by the Oxford Clinical Trials Research Unit (OCTRU). There is no randomisation to different interventions as part of the MONITOR-PsA cohort. Randomisation for any embedded trials is undertaken via the same centralised service (RRAMP) producing computer-generated randomised allocations using a minimisation approach including a random element, which will ensure balanced allocations across the treatment groups.

### Concealment mechanism {16b}

No randomisations are performed as part of the MONITOR-PsA cohort; as such no allocation concealment applies to this study. Embedded trials will randomise participants to their intervention with appropriate allocation concealment. Details of these will be reported in the protocols for the relevant trials.

### Implementation {16c}

**Fig. 1:**
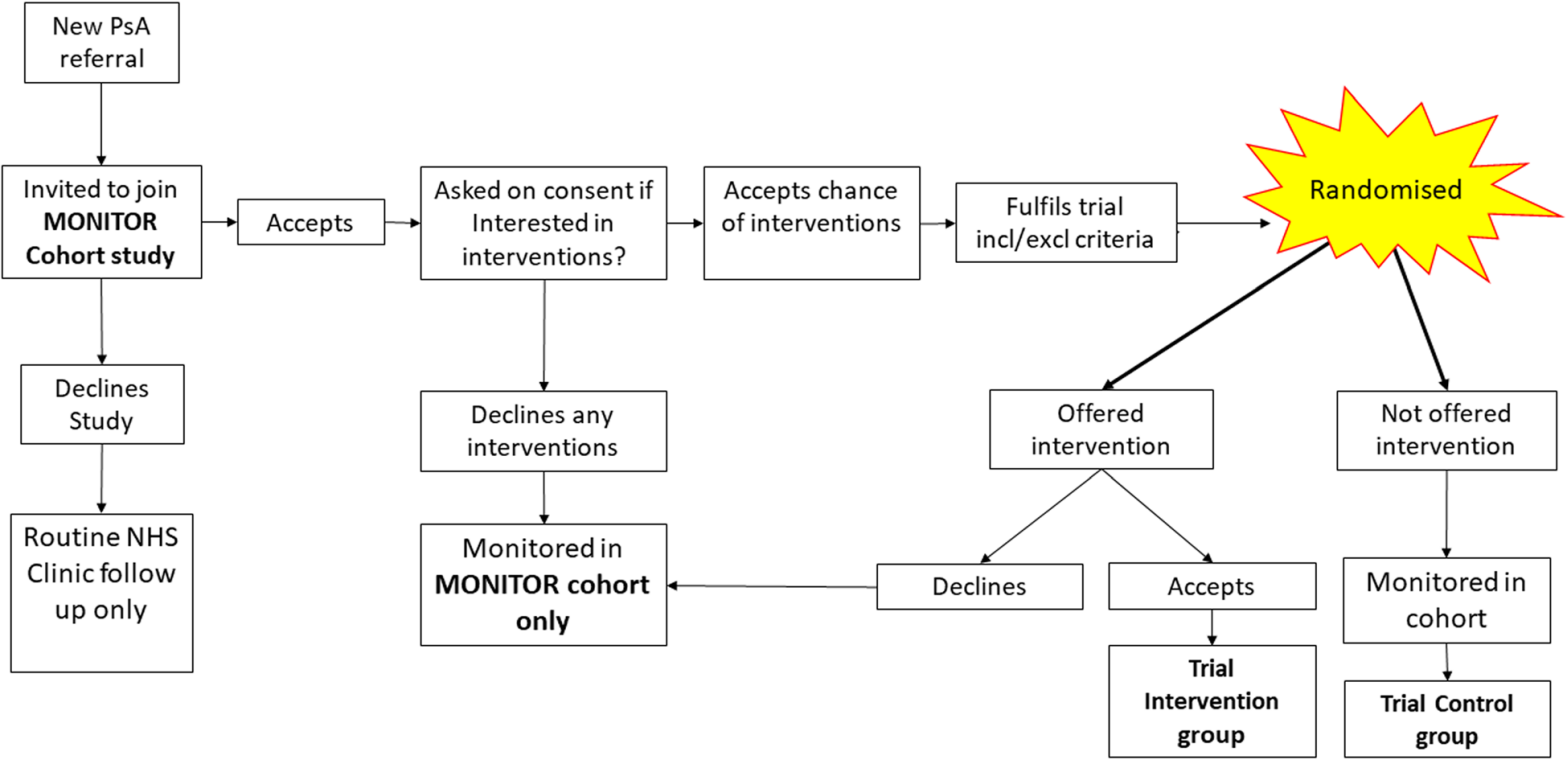
Schematic of multi-stage consent process

Participants will be registered in the MONITOR-PsA cohort using the central service (RRAMP) run by OCTRU. If patients have consented in the MONITOR-PsA consent form to be contacted about and randomised into embedded studies, then they will be evaluated for inclusion in any active trials at the appropriate treatment time point. This is aided by a reminder within the cohort database which identifies potentially eligible participants and highlights this to the clinical team. Due to the TWiCs design, randomisation occurs before all eligibility criteria for embedded trials can be confirmed. This is because participants randomised to standard care will not undergo further consent procedures and it allows some trial specific procedures to be limited to the relevant study arms (for example, screening for HIV and hepatitis prior to the use of biologics). To allow this early randomisation, with subsequent confirmation of eligibility, we have implemented a two-stage randomisation process outlined in Fig. 1.

If a participant is believed to be eligible for one of the embedded trials, then the process of initial or first stage randomisation will be performed by the study team using the RRAMP system. Initial assessment of inclusion/exclusion criteria that do not require blood tests or other invasive assessments is performed by the study team. If a patient appears to be eligible, they are randomised and allocated to a treatment group. Patients randomised to standard care do not receive any additional information about the embedded study and are given treatment as usual within the cohort. Participants randomised to an intervention are then given a specific patient information leaflet for the intervention they have been randomised to and invited to join the study. If they decline, they are treated as usual via the cohort. If they consent, further investigations are performed as necessary for that treatment arm and then a second stage randomisation is completed allowing assessment of all inclusion and exclusion criteria.

## Assignment of interventions: blinding

### Who will be blinded {17a}

Within the cohort, all participants receive open-label treatment via usual care pathways. Within embedded trials, treatment is usually open-label. To minimise bias and ensure fair assessment of outcome measures, a blinded assessor such as a study nurse performs the clinical assessments at each study visit. To ensure that blinding is maintained within the embedded trials, the assessments are performed within the database in three distinct sections, each with restricted with password controls. Firstly, the participant completes their questionnaires, then the assessor enters a password to access the clinical assessments, and finally the clinician enters a separate password to enter information about side effects, drug prescriptions and health utilisation data. The final screen summarises key outcomes including the MDA, PASDAS score, BASDAI and PsAID to support clinical decision making.

### Procedure for unblinding if needed {17b}

Not applicable, as participants and treating clinicians are not blinded to the intervention in this cohort study.

## Data collection and management

### Plans for assessment and collection of outcomes {18a}

The primary outcome, the PASDAS response, is a composite score including patient-reported outcomes, clinical assessments and a laboratory C-reactive protein (CRP) value. All data entry is done directly into the clinical database (OpenClinica) via tablet computers using the study number as an identifier. Patient-reported outcomes are also collected electronically wherever possible.

Clinical assessments of disease activity are performed by the blinded assessor who has undergone appropriate training in the assessments required including joint counts, enthesitis counts and Psoriasis Area and Severity Index (PASI) scores. The GRAPPA training modules for clinical assessments have been used to train new staff. As with the questionnaires, these are inputted directly into the study database via a tablet computer. All fields must be completed on each page of the electronic CRFs to minimise missing data and range checks are in place for all numeric fields to promote data quality. As blood tests are often not available until shortly after the appointment, these can be entered later into the study database, but an estimated PASDAS is calculated on the day of the appointment using the most recent CRP result.

The individual outcomes collected have been carefully chosen to cover all of the new 2016 Outcome Measures in Rheumatology (OMERACT) core and strongly recommended domains for PsA studies [23]. All of these outcomes have been validated in PsA and PASDAS was chosen as the primary outcome as it encompasses multiple domains of PsA [24]. A good response level (reduction from baseline of ≥ 1.6 and final score of ≤ 3.2) [18] has been chosen as this level of response is desirable for physicians and patients [18]. This is different to the TICOPA primary outcome but data are available for comparison [6, 25].

### Plans to promote participant retention and complete follow-up {18b}

Study visits in the cohort are deliberately aligned to usual clinical care. Given that most DMARD therapies can take up to 12 weeks to work, assessment in early arthritis is often at approximately 3-month intervals. Once patients are stable, appointments are generally gradually spaced out to minimise disruption to patients’ lives. Therefore, follow-up will be every 12 weeks in year one, every 6 months in year 2 and annually thereafter. If participants change therapy later in the course of their disease, additional study visits will be performed at weeks 12, 24 and 48 after any treatment change. This will allow development of embedded trials testing alternative treatment strategies in more established disease.

Following the development of the COVID-19 pandemic in 2020, some remote follow-up had to take place to ensure ongoing safety on treatment and subsequently this was permitted (particularly at week 36) under a specific COVID-19-related amendment. The primary endpoint of the study requires physical assessments by a clinician but during lockdown, limited information was collected via remote follow-up when physician visits were not possible.

As the cohort itself is non-interventional, participants who withdraw will withdraw completely, including withdrawal from follow-up. If they consent, their data up to the point of withdrawal will be retained and utilised in any analysis. Participants in embedded trials will be able to withdraw from the intervention protocols but remain in the cohort for follow-up.

### Data management {19}

All information concerning the data management procedures of the MONITOR-PsA cohort and embedded studies are included in study specific data management plans. Access to the study database is limited to authorised personnel using individual log-ins. The study database is stored on secure University of Oxford servers with appropriate backup. In some other study sites, data are collected using their local secure databases supporting existing database projects, before being transferred securely to Oxford for amalgamation.

To ensure high-quality data, databases also include multiple data validations, including checks for plausible ranges, and valid date ranges. Additional data checks are routinely performed by the study statistician. The calculation of composite scores will be verified before any analysis.

### Confidentiality {27}

All data will be processed in compliance with applicable data protection legislation, and all documents will be stored safely in confidential conditions. On all study-specific documents, other than the signed ICF and patient contact details, the participant will be referred to by the study participant number/code, not by name. Identifiable contact information will be stored separately from study data and held at the local research site and by the central coordinating team. For central monitoring purposes, completed consent forms will be reviewed by the coordinating trials unit, but these will be transferred via secure data transfer to ensure personal details are secure at all times.

### Plans for collection, laboratory evaluation and storage of biological specimens for genetic or molecular analysis in this trial/future use {33}

Routine safety and efficacy blood tests will be performed for all patients receiving disease-modifying therapy as done in standard NHS care, with samples handled according to local practice. The results of the routine NHS blood tests will be recorded to monitor safety of the treatment, disease activity and for the study, to calculate composite measures of efficacy. Participants consenting to additional biological sampling will be asked to provide up to 120 ml of blood at each study visit. This would be taken at the same time as laboratory blood tests performed in the clinic for safety and efficacy monitoring as part of standard care.

Participants may also be asked to give samples of urine and stool at these visits. Urine samples would be collected in clinic, participants will provide stool samples in their own home using containers provided during their clinic visit and these can be delivered to the sites at a visit or sent in the post. In some circumstances, participants will also be asked to consent to the collection of surplus samples of synovial fluid where available. Synovial fluid samples would only be taken at the time of arthrocentesis for diagnostic and therapeutic reasons. Up to 100 ml is usually aspirated from a large joint such as a knee, only 10mls of which is used for diagnostic culture and microscopy, the remainder being surplus to clinical requirements. If synovial membrane were removed during diagnostic or therapeutic arthroscopy, a small amount (less than 50%) would be studied, once samples had been sent for histology. This material would otherwise be discarded as surplus to clinical requirements.

Serum and synovial fluid will be frozen for analysis of constituents including proteins by enzyme-linked immunosorbent assay (ELISA), electrophoresis, mass spectrometry and other biochemical assays. Mononuclear cells will be obtained by density gradient centrifugation separation from blood and synovial fluid. Immunophenotyping by flow cytometry will be carried out at baseline and in-vitro assays will be performed. The remainder of cells will be frozen for batched experiments. DNA and RNA will be extracted for typing of molecules of immunological importance including human leukocyte antigen typing and transcriptomic analysis. Stool and/or urine samples will be studied for biomarkers including genetic and metabolomics analysis of microorganisms potentially influencing the immune system.

In the future, participants may be invited to participate in subsequent ethically approved embedded clinical trials based on phenotyping or genetic data obtained through the biological sampling. These data will be held in a pseudonymised form and used to assess eligibility for future studies where a participant has consented to this. The link to participant’s identifiable information and contact details will be held by the study team and used only where a participant has consented to be contacted about future research. Any future contact would come from this research team and would make clear participants are under no obligation to participate in any such study. Samples will be stored frozen in secure research facilities for the duration of the study. Samples will only be identifiable by the participant’s study code.

## Statistical methods

### Statistical methods for primary and secondary outcomes {20a}

Analysis will be undertaken within OCTRU, supervised by the statistician for the study and will be performed after approximately 200 participants receiving the standard treatment have been followed up for 48 weeks. All analyses will focus on the participants not included in any of the interventional arms of the embedded trials.

The primary endpoint is the proportion of patients achieving the PASDAS ‘good’ response at 48 weeks, as defined in {12}. The primary analysis will report the outcomes together with 95% confidence intervals. The estimates for the participants receiving the standard treatment (pragmatic ‘step-up’ ‘treat to target’ treatment) in MONITOR-PsA will be compared informally to the outcomes for the relevant trial arms of the TICOPA trial (i.e. no formal statistical tests will be performed).

Secondary endpoints of the MONITOR-PsA cohort study will be presented descriptively for each follow-up time point, including means with 95% confidence intervals and/or medians with interquartile ranges and ranges for continuous variables, proportions and corresponding 95% confidence intervals for binary outcomes.

Analyses of the participants recruited to the embedded trials will be outlined in the separate protocols and statistical analysis plans, and any deviations from the plans will be justified and reported in the final report and publications.

### Interim analyses {21b}

No formal interim analyses are planned for the MONITOR-PsA cohort ahead of the primary analysis. The Data Safety and Monitoring Committee may see interim descriptive summaries of accumulating data in confidence.

Additional analyses of the longer-term follow-up are planned to take place after the primary analysis of this cohort. Subsets of the cohort including participants enrolled in the embedded trials may also be analysed throughout the duration of the cohort.

### Methods for additional analyses (e.g. subgroup analyses) {20b}

Supplementary analyses will be undertaken to explore the many baseline prognostic factors in order to examine which patients most benefit from this treatment and whether important subgroups can be defined. These analyses will be exploratory and utilise descriptive summaries and logistic regression models.

### Methods in analysis to handle protocol non-adherence and any statistical methods to handle missing data {20c}

Missing data will be minimised by careful study conduct and data management. Missing data will be described with reasons given where available; the number and percentage of individuals in the missing category will be presented.

The nature and mechanism for missing variables and outcomes will be investigated, and if appropriate multiple imputation will be used. Sensitivity analyses will be undertaken assessing the underlying missing data assumptions, if appropriate.

### Economic analysis

An economic evaluation, in the form of a cost-effectiveness analysis, is not planned as part of the MONITOR PsA study. Instead, the analysis will be presented in a descriptive and informative way so as to establish comparisons in costs and QALYs estimates with the predecessor of MONITOR, the TICOPA trial.

Data will be collected on the health resources used by participants during the study period. At 24, 48 and 96 weeks post-randomisation participants and health professionals will be asked to complete economic questionnaires in relation to hospital admission, medication, outpatient visits and community health care. Unit cost data will be obtained from national databases such as the BNF [26] and PSSRU costs of health and social care [27].

Health Related Quality of life will be estimated using the EuroQol EQ-5D 5 L for self-completion at baseline, 24, 48 and 96 weeks post-randomisation. As per the NICE position statement, the responses to the EQ-5D-5L will be converted into multi-attribute utility scores using the approved ‘cross-walk’ to the 3 L instrument using the mapping function developed by van Hout et al. [28] and the converted responses will be valued using the established time trade-off utility algorithm for the UK [29]. QALYs will be calculated as the area under the utility curve of utility scores from baseline, 24, 48 and 96 weeks, data using the trapezoidal rule [30].

The economic analysis will be conducted from a UK NHS and Personal Social Services perspective (PSS) using the cohort data.

### Plans to give access to the full protocol, participant level-data and statistical code {31c}

The full study protocol can be accessed by contacting the trial team at MonitorPsA@ndorms.ox.ac.uk. Anonymised participants level data and statistical codes used for the analysis are available on request and subject to data sharing agreements. Data sharing may only be possible after relevant embedded trials have been published.

## Oversight and monitoring

### Composition of the coordinating centre and trial steering committee {5d}

This study will be conducted as part of the portfolio of trials in the registered UKCRC Oxford Clinical Trials Research Unit (OCTRU) at the University of Oxford. It will follow their Standard Operating Procedures ensuring compliance with the principles of Good Clinical Practice and the Declaration of Helsinki and any applicable regulatory requirements. The day-to-day management of the study will be the responsibility of the Clinical Trials Coordinator. This will be overseen by the Study Management Group, who will meet monthly to assess progress, as well as by OCTRU senior staff. It will be the responsibility of the Clinical Trials Coordinator and CI to undertake training of the research staff at each of the study centres. The study statistician and health economist will be closely involved in setting up data capture systems, design of databases and clinical reporting forms. A Trial Steering Committee (TSC) and an Independent Data & Safety Monitoring Committee (DSMC) have been established to cover this cohort study and any embedded trials which includes patient research partners.

The TSC, which includes independent members, provides overall supervision of the study on behalf of the funder. Its terms of reference have been agreed with the funder and drawn up in a TSC charter which will outline its roles and responsibilities. Meetings of the TSC will take place at least once a year during the recruitment period.

An outline of the remit of the TSC is to:

- Monitor and supervise the progress of the study towards its interim and overall objectives,
- Review at regular intervals relevant information from other sources,
- Consider the recommendations of the DSMC,
- Inform the funding body on the progress of the study.

### Composition of the data monitoring committee, its role and reporting structure {21a}

The DSMC is a group of independent experts external to the study who assess the progress, conduct, participant safety and, if required critical endpoints of a clinical trial. The DSMC has agreed and adopted an appropriate charter that defines its terms of reference and operation in relation to oversight of the study. They will not be asked to perform any formal interim analyses of effectiveness. They will, however, review accruing data, summaries of the data presented by treatment group, and will assess the screening algorithm against the eligibility criteria. They will also consider emerging evidence from other related research and review related SAEs that have been reported. They may advise the chair of the Trial Steering Committee at any time if, in their view, the study (or embedded trial) should be stopped for ethical reasons, including concerns about participant safety. DSMC meetings will be held at least annually during the recruitment phase of the study. Full details including names will be included in the DSMC charter. The TSC and DSMC for the study will also cover any embedded trials within the cohort to allow cohesive review of any safety issues.

### Adverse event reporting and harms {22}

As the cohort supports clinical trials of investigational medicinal products (IMPs), considerable thought has been given to safety reporting. Within the cohort, only serious adverse events (SAE) occurring to a participant where in the opinion of the CI the event was ‘related’ (resulted from administration of any of the research procedures) and ‘unexpected’ in relation to those procedures will be reported. However, for participants in the embedded trials, SAEs will be reported according to the individual trial protocols.

All non-serious adverse events will not be collected in the cohort or current trials as all the IMPs are licenced, currently used in PsA and have well documented safety profiles. Only adverse events of special interest (either extra-articular manifestations of the disease or those likely to be related to the therapy used for PsA) occurring during the study will be collected at each study visit by patient questionnaire and physician report.

The following information will be recorded: severity, assessment of relatedness to trial medication and action taken. The severity of events will be assessed on the following scale: 1 = mild, 2 = moderate, 3 = severe. Follow-up information will be provided as necessary.

### Frequency and plans for auditing trial conduct {23}

The study may be monitored, or audited in accordance with the current approved protocol, Good Clinical Practice (GCP), relevant regulations and standard operating procedures. A Monitoring Plan will be developed according to OCTRU’s standard operating procedures (SOPs) which involves a risk assessment. The monitoring activities are based on the outcome of the risk assessment and may involve central monitoring and on-site monitoring.

The study coordinator will be responsible for ensuring adherence to the trial protocols at the trial sites. Quality assurance checks will be undertaken by OCTRU to ensure integrity of study entry procedures and data collection. OCTRU has a quality assurance team who will audit this study by conducting (at least once in the lifetime of the study, more if deemed necessary) review of the Trial Master File (TMF). Furthermore, the processes of consenting, registration, provision of information and provision of treatment will be monitored. Written reports will be produced for the TSC, informing them if any corrective action is required.

### Plans for communicating important protocol amendments to relevant parties (e.g. trial participants, ethical committees) {25}

Any significant amendments to the protocol will be reviewed by the sponsor and the approving ethics committee prior to any implementation. If any new safety data becomes available, or there is a significant change to the study design that would impact on patients, then participants would be given an updated patient information leaflet and where appropriate asked to reconsent.

### Dissemination plans {31a}

Trial results will be disseminated via scientific publications and conference presentations. Results will also be posted via the ClinicalTrials.gov website. With assistance from the patient research partners, a lay summary of the results will be developed for dissemination to patients and the public. The Investigators and research study team will be involved in reviewing drafts of the manuscripts, abstracts, press releases and any other publications arising from the study. Authorship will be determined in accordance with the ICMJE guidelines and other contributors will be acknowledged. Authors will acknowledge that the study was funded by the NIHR.

## Discussion

The TICOPA trial has proven the benefits of a treat-to-target approach but translation into clinical practice is required to ensure that clinical care is optimised. The lack of a feasible cost-effective model for this treat to target approach has been an issue in widespread adoption of the approach. The MONITOR-PsA cohort will provide real world evidence of the effectiveness of a feasible treat to target model with 3 monthly regular review.

All international treatment recommendations have supported the treat to target concept but have concluded that there is a lack of evidence to support specific therapeutic choices in PsA. Within TICOPA all of the patients were treated according to the same step up protocol using DMARDs and then biologics. Given the heterogeneous nature of the disease, it seems likely that this approach could be optimised by personalising therapeutic choices to the individual within the T2T approach. When designing the study, the aim was to provide a platform for testing of different forms of personalised medicine in future studies including those based on disease severity, a domain-based approach or a focus on prognosis. For this reason the TWiCs study design was adopted.

Since the first publication on the cohort multiple RCT or TWiCs trial in 2015, it has been adopted for a number of studies in the last few years. However it remains a relatively new study design. When developing the study initially, we considered whether it would be more appropriate to have one single unified protocol covering the cohort and any embedded trials. The sponsor’s office at the University of Oxford had no prior experience of the TWiCS design but suggested using a template allowing nested sub-studies. This would have been efficient as outcomes and time points have to be identical between the cohort and any trials. It would also allow any amendments to be implemented within one protocol. However, embedding trials as sub-studies of the main cohort protocol would have meant that the trials would never officially close given the single EudraCT registration that would be provided under European regulations. Nearly all previous TWiCs studies have evaluated other therapies such as physiotherapy and radiotherapy rather than including interventional drug trials. Particularly with a drug trial, this would have caused issues in the future as the trials cannot officially be closed. For this reason, we chose to have separate protocols for the cohort and the embedded studies, allowing any embedded trials to be added and related to the cohort, but then closed when they are complete. The downside to this is that any change to the study design necessitates amendments to multiple protocols with multiple sponsor and ethics committees reviews with associated additional workload, delays and costs.

The inclusion of controlled trials of investigational medicinal products presents specific challenges as approval is required from the Medicines Health and Regulatory Authority (MHRA) in the UK and they must ensure that good clinical practice is followed. To our knowledge, only one pilot TWiCs drug trial has been established testing treatments for mesothelioma [16]. The particular concern in this case is that participants are randomised to different treatment arms of a drug trial without any further consent after the cohort consent [31]. For that reason, input was sought from a specialist at the MHRA despite the fact that the cohort itself is non-interventional. The key is to ensure that the consent form of this observational study would also satisfy requirements for subsequent drug trials. Advice from the MHRA on wording was invaluable in this regard.

The consent process required for this design also mandated changes to the usual randomisation procedures. Randomisation must be performed early in the process prior to additional consent from participants. At this time in the process, additional study specific procedures cannot be performed to confirm all inclusion/exclusion criteria until the randomisation arm is known. For this reason, the randomisation has been split into two stages. The first stage allows assessment of basic clinical inclusion criteria and allows randomisation to one of the study arms. Following a full baseline assessment, including trial consent and baseline blood tests, the study team must confirm the randomisation in a second stage to ensure that all inclusion/exclusion criteria are met before participants embark on study mandated treatment.

The use of a novel trial design in this cohort, has identified challenges and required the study team to actively engage stakeholders, such as the sponsor, the MHRA and support teams within the clinical trials unit with education, discussion and development of novel solutions. However, solutions have been identified to cover all of the key issues raised here and the cohort is now successfully established in multiple sites in the UK. We hope that in the long term, this cohort can supply important insights into the natural history of early PsA, whilst simultaneously supporting a number of embedded pragmatic trials providing an evidence base of optimal treatment approaches in routine clinical practice.

## Trial status

The study is open for inclusion under protocol version 6, 28 July 2020. Recruitment started in April 2018 and recruitment of the initial 500 patients will likely be completed in 2023.

## Data Availability

Access to the data for purposes of research other than this study, would be at the discretion of the CI and in accordance with the terms of the patient consent as per the patient information sheet and consent form. At this point in time, there is no prospective data sharing planned although data sharing may be possible with appropriate approvals and data may be transferred to a repository in accordance with the protocol and REC approval.

## Abbreviations

BASDAI: Bath ankylosing spondylitis disease activity index
BSR: British Society of Rheumatology
CASPAR: Classification of Psoriatic arthritis
CI: Chief investigator
cmRCT: Cohort multiple randomised controlled trial
CRF: Case report form
CRP: C-reactive protein
DMARD: Disease-modifying anti-rheumatic drug
DSMC: Data and safety monitoring committee
eCRF: Electronic case report form
EULAR: European Alliance of Associations for Rheumatology
GCP: Good clinical practice
GRAPPA: Group for Research and Assessment of Psoriasis and Psoriatic Arthritis
ICF: Informed consent form
ICMJE: International Committee of Medical Journal Editors
IL: Interleukin
IMPs: Investigational medicinal products
JAK: Janus kinase
MDA: Minimal disease activity
MHRA: Medicines Health and Regulatory Authority
MONITOR-PsA: Multicentre Observational Initiative in Treat to target Outcomes in Psoriatic Arthritis
NHS: National Health Service
NICE: National Institute of Health and Care Excellence
NIHR: National Institute of Health Research
OCTRU: Oxford Clinical Trials Research Unit
OMERACT: Outcome Measures in Rheumatology
PASDAS: Psoriatic arthritis disease activity score
PASI: Psoriasis area and severity index
PCS: Physical component score
PDE4: Phosphodiesterase 4
PI: Principal investigator
PIS: Patient information sheet
PsA: Psoriatic arthritis
PsAID: Psoriatic arthritis impact of disease
PsARC: Psoriatic arthritis response criteria
QALY: Quality adjusted life year
RA: Rheumatoid arthritis
RCT: Randomised controlled trial
RRAMP: Registration and randomisation and management of product
SAE: Serious adverse event
SOP: Standard operating procedure
SpA: Spondyloarthritis
T2T: Treat-to-target
TICOPA: Tight control of psoriatic arthritis
TMF: Trial master file
TNF: Tumour necrosis factor
TSC: Trial steering committee
TSQM: Treatment satisfaction questionnaire for medication
TWiCs: Trials within cohorts
VAS: Visual analogue scale
WPAI: Work productivity and activity impairment questionnaire
